# Norhierridin B, a New Hierridin B-Based Hydroquinone with Improved Antiproliferative Activity

**DOI:** 10.3390/molecules25071578

**Published:** 2020-03-30

**Authors:** Pedro Brandão, Joana Moreira, Joana Almeida, Nair Nazareth, Ivo E. Sampaio-Dias, Vitor Vasconcelos, Rosário Martins, Pedro Leão, Madalena Pinto, Lucília Saraíva, Honorina Cidade

**Affiliations:** 1 Laboratory of Organic and Pharmaceutical Chemistry, Department of Chemical Sciences, Faculty of Pharmacy, University of Porto, Rua de Jorge Viterbo Ferreira 228, 4050-313 Porto, Portugal; pedrocgbrandao@gmail.com (P.B.); joana.m26@hotmail.com (J.M.); madalena@ff.up.pt (M.P.); 2 CIIMAR/CIMAR, Interdisciplinary Centre of Marine and Environmental Research, University of Porto, Edifício do Terminal de Cruzeiros do Porto de Leixões, Avenida General Norton de Matos, S/N, 4450-208 Matosinhos, Portugal; vmvascon@fc.up.pt (V.V.); mrm@ess.ipp.pt (R.M.); pleao@ciimar.up.pt (P.L.); 3 LAQV/REQUIMTE, Laboratory of Microbiology, Department of Biological Sciences, Faculty of Pharmacy, University of Porto, Rua Jorge Viterbo Ferreira, 228, 4050-313 Porto, Portugal; JoanaAlmeida15@gmail.com (J.A.); naircampos@gmail.com (N.N.); 4 LAQV/REQUIMTE, Department of Chemistry and Biochemistry, Faculty of Sciences, University of Porto, Rua do Campo Alegre 687, 4169-007 Porto, Portugal; idias@fc.up.pt; 5 Department of Biology, Faculty of Sciences, University of Porto, Rua do Campo Alegre, Edifício FC4, 4169-007 Porto, Portugal; 6 Health and Environment Research Centre, School of Health, Polytechnic Institute of Porto, Rua Dr. António Bernardino de Almeida, 400, 4200-072 Porto, Portugal

**Keywords:** antitumor activity, hierridin B, hydroquinones, quinones

## Abstract

Hierridin B (**6**), a methylated hydroquinone isolated from the marine picocyanobacterium *Cyanobium* sp. LEGE 06113, moderately inhibited the growth of colon adenocarcinoma HT-29 cells. Aiming to improve the potential antitumor activity of this natural product, the demethylated analogue, norhierridin B (**10**), as well as its structurally-related quinone (**9**), were synthesized and evaluated for their growth inhibitory effect on a panel of human tumor cell lines, including the triple-negative breast cancer (TNBC) cells MDA-MB-231, SKBR3, and MDA-MB-468. Norhierridin B (**10**) showed a potent growth inhibitory effect on all cancer cell lines. Moreover, the growth inhibitory effect of compound **10** on MDA-MB-231 cells was associated with cell cycle arrest and apoptosis. Norhierridin B (**10**) interfered with several p53 transcriptional targets, increasing p21, Bax, and MDM2, while decreasing Bcl-2 protein levels, which suggested the potential activation of a p53 pathway. Altogether, these results evidenced a great improvement of the antitumor activity of hydroquinone **10** when compared to **6** and its structurally-related quinone (**9**). Notably, hydroquinone **10** displayed a prominent growth inhibitory activity against TNBC cells, which are characterized by high therapeutic resistance.

## 1. Introduction

**Figure 1:**
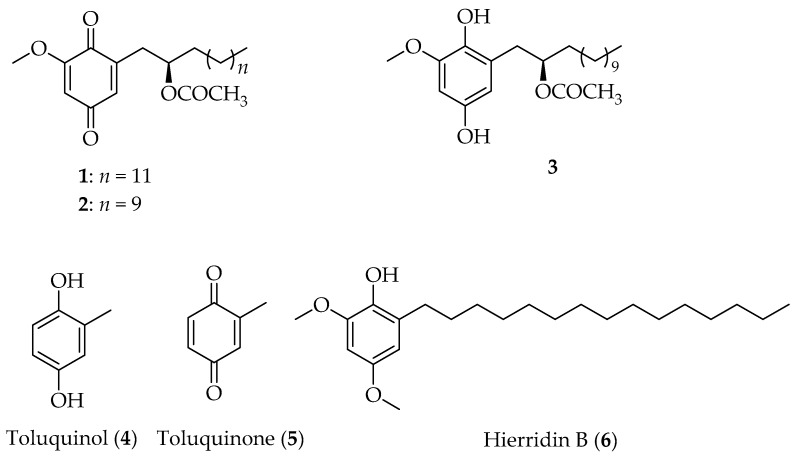
Structure of quinones and hydroquinones with cytotoxic activity.

Quinones/hydroquinones constitute a family of metabolites, widespread in nature, with a wide range of biological activities, including cytotoxicity to cancer cells [1,2,3]. Particularly, alkyl 1,4-benzoquinones/hydroquinones with aliphatic side chains have been found attractive for their in vitro growth inhibitory effect against several human cancer cell lines [3,4] (Figure 1). Among these secondary metabolites, a wide array of bioactive compounds has been isolated from nature, namely from plant species and marine organisms. For instance, compounds **1**–**3** (Figure 1), isolated from *Ardisia virens*, exhibited a potent inhibitory effect against breast adenocarcinoma MCF-7, non-small cell lung cancer NCI-H460, and glioblastoma SF-268 cell lines (concentration that caused cell growth inhibition of 50%, GI_50_, of 2.21–11.32 µM) [4], with hydroquinone **3** being more potent than quinone **2**. Toluquinol (**4**) (Figure 1), isolated from the marine fungus *Penicillium* sp. HL-85-ALS5-R004, is another example of a natural product with a promising in vitro growth inhibitory effect in breast adenocarcinoma MDA-MB-231 and glioblastoma U87-MG cells, as well as in promyelocytic leukemia HL-60, fibrosarcoma HT-1080, and colorectal adenocarcinoma HT-29 cell lines [5]. Interestingly, when comparing the in vitro growth inhibitory activity of quinones with structurally related hydroquinones, it seems that the presence of a hydroquinone could be more favorable for activity. In fact, the IC_50_ values obtained for quinone **2** are slightly higher than those of the structurally related hydroquinone **3** [4]. Moreover, toluquinone (**5**), a synthetic analogue of the marine hydroquinone toluquinol (**4**), showed lower in vitro growth inhibitory activity against a panel of cancer cell lines of breast adenocarcinoma (MDA-MB-231, GI_50_ of 3.4 ± 0.1 µM), promyelocytic leukemia (HL-60, GI_50_ of 5.6 ± 1.9 µM), glioblastoma (U87-MG, GI_50_ of 8.8 ± 0.9 µM), fibrosarcoma (HT-1080, GI_50_ of 4.9 ± 1.7 µM), and colorectal adenocarcinoma (HT-29, GI_50_ of 9.5 ± 0.5 µM) than toluquinol (**5**) (MDA-MB-231: GI_50_ of 2.3 ± 0.8 µM, HL-60: GI_50_ of 1.7 ± 0.5 µM, U87-MG: GI_50_ of 5.6 ± 1.5 µM, HT-1080: GI_50_ of 1.4 ± 0.6 µM and HT-29: GI_50_ of 4.1 ± 0.4 µM), further supporting that the presence of a hydroquinone is favorable for the antitumor activity [5].

Recently, following the search for new bioactive secondary metabolites from marine cyanobacteria, we identified hierridin B (**6**), a methylated hydroquinone derivative with a C15 aliphatic chain isolated from the marine picocyanobacterium *Cyanobium* sp. LEGE 06,113 [6]. Compound **6** moderately inhibited the growth of colon adenocarcinoma HT-29 cell line, with a GI_50_ of 100.2 μM [6]. In order to obtain new structurally-related quinone/hydroquinone with improved antiproliferative activity in cancer cell lines, the demethylated hierridin B derivative, norhierridin B (**10**), as well as structurally-related quinone **9** and compound **6,** were synthesized and evaluated for their growth inhibitory effect in a panel of human tumor cell lines.

## 2. Results and Discussion

### 2.1. Synthesis

**Scheme 1:**
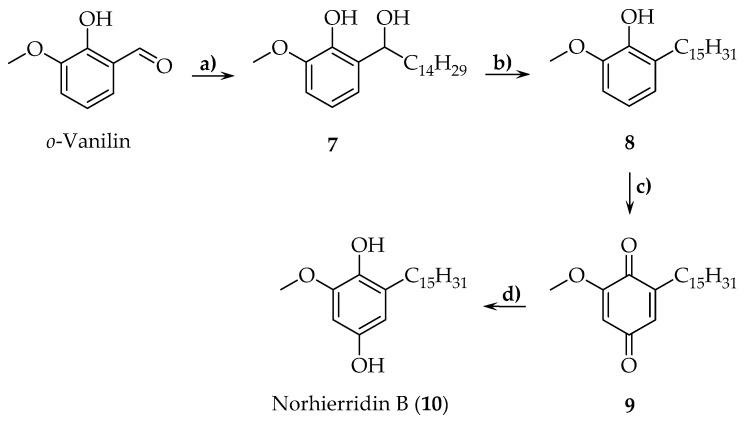
Synthesis of norhierridin B (10). (a) C_14_H_29_Br, Mg, Et_2_O, 35 °C, 2 h; (b) HCOOH, Pd/C, EtOH/H_2_O, 80 °C, 4 h; (c) O_2_, salcomine, DMF, r. t., 24 h; (d) Na_2_S_2_O_4_, CHCl_3_/H_2_O, r.t., 10 min.

Norhierridin B (**10**) was synthesized according to the strategy illustrated on Scheme 1. In order to explore the growth inhibitory effect of demethylated hierridin B (**6**) derivative (**10**), as well as of its 1,4-benzoquinone analogue (**9**), a synthetic pathway was planned in order to obtain this analogue as an intermediate. Firstly, Grignard addition to *o*-vanilin using tetradecyl magnesium bromide in situ prepared from Mg turnings and tetradecyl bromide in dry diethyl ether afforded the corresponding carbinol **7** with 14% yield. The reduction of α-hydroxy group was firstly attempted by hydrogenolysis catalyzed by palladium on carbon (Pd/C) using sulfuric acid as hydrogen donor, as reported by González et al. [7]. Nevertheless, as no reaction occurred, a similar reaction with Pd/C using formic acid as hydrogen donor was conducted, affording the reduced derivative **8** with 93% yield. The high yield obtained reinforces the advantage of using palladium catalysts and formic acid in the catalytic transfer hydrogenolysis of benzylic alcohols, as described previously [8,9,10]. Phenol **8** was oxidized by streaming oxygen into a solution of DMF containing salcomine as catalyst, affording the 1,4-benzoquinone **9** with good yield (82%). The reduction of quinone **9** using Na_2_S_2_O_4_ as a reducing agent afforded norhierridin B (**10**) with 87% yield.

The structure elucidation of the new hydroquinone **10** was established by NMR and HRMS (Figures S4 and S5). The newly synthesized quinone **9**, as well as intermediates **7** and **8,** were characterized by NMR techniques, and data were in accordance with that previously reported [11,12]. ^13^C-NMR assignments were determined by 2D heteronuclear single quantum correlation (HSQC) and heteronuclear multiple bond correlation (HMBC) experiments. Hierridin B (**6**) was synthesized and characterized as described previously [13].

### 2.2. Biological Activity

Tumor Cell Growth Inhibitory Activity Evaluation

The growth inhibitory effect of norhierridin B (**10**), as well as of its structurally related 1,4-benzoquinone (**9**), intermediates **7** and **8,** and hierridin B (**6**), was evaluated in a panel of distinct human cancer cell lines of melanoma (A375), hepatocarcinoma (Huh-7), colon (HCT116), and triple-negative breast cancer (TNBC) (MDA-MB-231, SKBR3, and MDA-MB-468), using the sulforhodamine (SRB) assay. A dose-response curve was obtained for each compound in the distinct cell lines and the concentration that induced half of growth inhibition (GI_50_) was determined after 48 h treatment (Table 1).

The obtained GI_50_ values revealed that all newly synthesized compounds (**7**, **8**, **9**, and **10**) had a much higher growth inhibitory activity than hierridin B (**6**) in all cancer cell lines (Table 1). Norhierridin B (**10**) was found to be the most effective against all cancer cell lines, exhibiting much lower GI_50_ values than its methyl derivative hierridin B (**6**). These results suggest that the presence of two *p*-hydroxy groups in the hydroquinone scaffold are important for the in vitro growth inhibitory activity. In addition, norhierridin B (**10**) displayed higher activity than its structurally related quinone (**9**), reinforcing that the presence of a hydroquinone scaffold is more favorable for the activity than a quinone scaffold, as already reported by Chang et al. [4] and Cheng-Sánchez et al. [5] for other structurally related hydroquinones/quinones.

**Figure 2:**
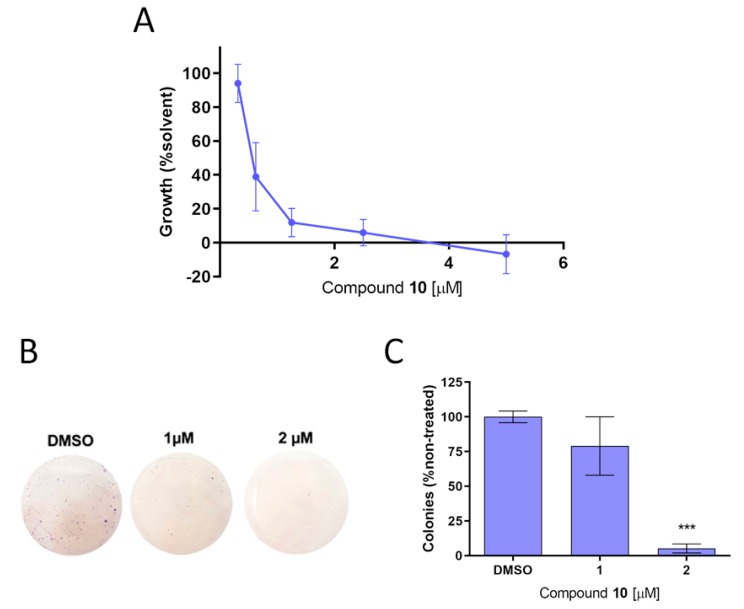
Norhierridin B (**10**) inhibits the growth of breast adenocarcinoma MDA-MB-231 cells. (**A**) Dose-response curve for the growth of MDA-MB-231 cells treated with compound **10** for 48 h, determined by SRB assay; data are mean ± SEM of four independent experiments; growth obtained with vehicle was set as 100%. **(B, C**) Colony formation assay for MDA-MB-231 cells treated with compound **10** for nine days: (**B**) images correspond to a representative experiment of two; (**C**) graph represents mean ± SEM of two independent experiments; values significantly different from DMSO: *** *p* < 0.001, unpaired Student’s *t*-test.

Interestingly, norhierridin B (**10**) exhibited a prominent growth inhibitory activity against TNBC cells MDA-MB-231, SKBR3, and MDA-MB-468, which are well-known by their high therapeutic resistance [14]. Moreover, an evident reduction of the norhierridin B (**10**) antiproliferative effect could be observed by SRB assay in non-tumorigenic MCF10A breast epithelial cell lines (10.36 ± 1.96 µM, three independent experiments). In fact, supporting the marked antiproliferative effect of compound **10** on MDA-MB-231 cells, observed by SRB assay (Figure 2A), a pronounced inhibition of the growth of these cancer cells was also observed at 2 µM by colony formation assay (Figure 2B,C).

**Figure 3:**
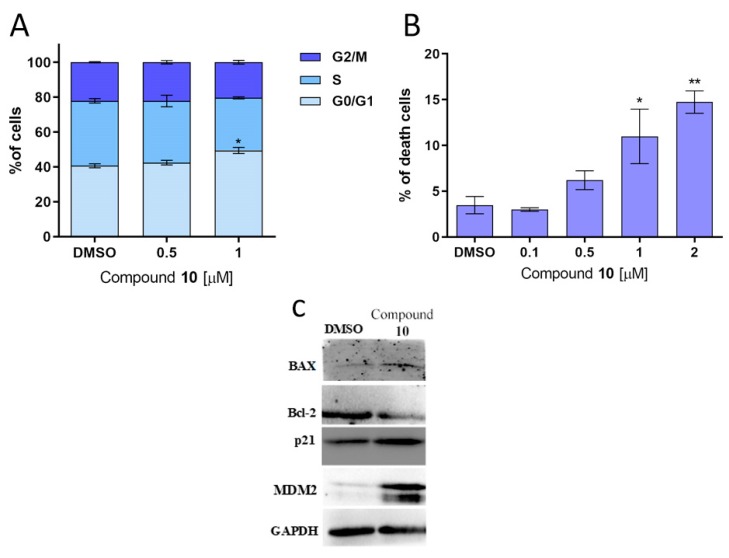
Norhierridin B (**10**) induces cell cycle arrest and death and interferes with p53 transcriptional targets. **(A**) Cell cycle progression was analyzed after 48 h treatment with compound **10** in MDA-MB-231 cells; data are mean ± SEM of three independent experiments; values significantly different from DMSO: *p < 0.05, unpaired Student’s *t*-test. **(B**) Percentage of death cells determined by trypan blue assay for MDA-MB-231 cells treated with compound **10** for 48 h; data are mean ± SEM of three independent experiments; values significantly different from DMSO: *p < 0.05, **p < 0.001, unpaired Student’s *t*-test. **(C**) Protein expression levels of p53 targets in MDA-MB-231 cells was analyzed by western blot after 48 h treatment with 2 µM compound **10**. Immunoblots are representative of three independent experiments; GAPDH was used as loading control.

It was also verified that the growth inhibitory effect of compound **10** on MDA-MB-231 cells was associated with the induction of apoptosis and cell cycle arrest. In fact, the 1 µM concentration of compound **10** caused a significant G0/G1-phase cell cycle arrest (Figure 3A), which was consistent with the visible increase of p21 protein expression levels observed by western blot analysis (Figure 3C). Moreover, by cell viability assay, 1 and 2 µM of compound **10** significantly increased the percentage of dead cells (Figure 3B), which was associated with an increase of the pro-apoptotic protein Bax and a decrease of the anti-apoptotic protein Bcl-2 (Figure 3C). It is interesting to note that compound **10** interfered with the protein expression levels of several p53 transcriptional targets, such as p21, Bax, Bcl-2, and MDM2 (Figure 3C), suggesting the activation of a p53 pathway in MDA-MB-231 cells. Collectively, these results indicate that compound **10** is very effective against TNBC cells. The poor prognosis and limited therapeutic options commonly observed with this type of cancer [14] render compound **10** worthy of further studies to confirm its promising application as potential anticancer agent, particularly against TNBC.

## 3. Material and Methods

### 3.1. Synthesis

All reactions were monitored by thin-layer chromatography (TLC). Purifications of compounds were carried out by flash chromatography using Macherey-Nagel silica gel 60 (0.04–0.063 mm). Melting points were obtained in a Köfler microscope (Wagner and Munz, Munich, Germany) and are uncorrected. ^1^H-NMR and ^13^C-NMR spectra were taken in CDCl_3_ at room temperature, on a Bruker Avance 300 instrument (300.13 MHz for ^1^H and 75.47 MHz for ^13^C, Bruker Biosciences Corporation, Billerica, MA, USA). Chemical shifts are expressed in ppm values relative to tetramethylsilane (TMS) used as an internal reference; ^13^C-NMR assignments were made by 2D (HSQC and HMBC) NMR experiments (long-range ^13^C-^1^H coupling constants were optimized to 7 Hz). HRMS mass spectrum was recorded as ESI (electrospray ionization) mode on a MicrOTOF spectrometer (Bruker Corporation, Billerica, MA, USA) at C.A.C.T.I.-University of Vigo, Spain. The commercially available reagents, and etoposide, were purchased from Sigma Aldrich Co. (Sintra, Portugal). Reagents and solvents were purified and dried according to the usual procedures described elsewhere [15]. Hierridin B (**6**) was synthesized as described previously [13]. The following materials were synthesized and purified by the described procedures.

#### 3.1.1. Synthesis of 2-(1-hydroxypentadecyl)-6-methoxyphenol (**7**)

Tetradecyl magnesium bromide was prepared from Mg turnings (2.01 g, 0.62 eq.) and tetradecyl bromide (37 g, 0.13 mol) in dry diethyl ether (200 mL) under a nitrogen atmosphere. *o*-Vanilin (10 g, 0.066 mol) was gradually added, and the mixture was then refluxed for 2 h, cooled, and poured into a saturated solution of NH_4_Cl (150 mL). The layers were separated, and the aqueous phase thoroughly extracted with diethyl ether (3 × 200 mL). The organic layer was washed with water, dried over anhydrous sodium sulfate, and evaporated under reduced pressure. The residue was purified by flash column chromatography (silica gel, *n*-hexane: ethyl acetate 8:2), affording **7** (3.23 g, 14%) as a white solid.

*2-(1-hydroxypentadecyl)-6-methoxyphenol* (**7**): ^1^H-NMR (CDCl_3_, 300.13 MHz): δ = 6.83–6.78 (m, 3H, H-3,-4,-5), 6.38 (s, 1H, 1-OH), 4.87 (t, *J* = 6.0 Hz, 1H, H-1′), 3.88 (s, 3H, 2-OCH_3_), 1.84–1.80 (m, 2H, H-2′), 1.30–1.24 (m, 24 H, H-3′-14′), 0.87 (t, *J* = 6.5 Hz, 3H, H-15′); ^13^C-NMR (CDCl_3_, 75.47 MHz) δ: 146.8 (C-2), 143.1 (C-1), 129.8 (C-6), 119.6 (C-4), 119.2 (C-5), 109.7 (C-3), 72.0 (C-1′), 56.0 (2-OCH_3_), 37.2 (C-2′), 31.9–22.7 (C-2′-14′), 14.1 (C-15′).

#### 3.1.2. Synthesis of 2-methoxy-6-pentadecylphenol (**8**)

In a 250 mL two neck round-bottomed flask equipped with a water-cooled condenser and a magnetic stirrer, ethanol (99 mL), water (20 mL), compound **7** (3.17 g, 9 mmol), formic acid (0.68 mL), and Pd/C 10% (*w*/*w*) (1.259 g) were added. The reactor was placed in a preheated oil bath and maintained at 80 °C under atmospheric pressure for 4 h. The reactor was allowed to cool down to r.t., and the ethanol was evaporated under reduced pressure. Water was added (20 mL), and the resulting solution was extracted with diethyl ether (3 × 100 mL). The organic layer was dried over anhydrous sodium sulfate and evaporated under reduced pressure affording a solid, which was crystallized from diethyl ether: methanol affording the title compound (**8**) (2,81 g, 93%) as a white solid.

*2-methoxy-6-pentadecylphenol* (**8**): ^1^H-NMR (CDCl_3_, 300.13 MHz): δ = 6.80–6.70 (m, 3H, H-3,-4,-5), 5.66 (s, 1H, 1-OH), 3.87 (s, 3H, 2-OCH_3_), 2.62 (t, *J* = 7.6 Hz, 1H, H-1′), 1.65–1.57 (m, 2H, H-2′), 1.27–1.25 (m, 24 H, H-3′-14′), 0.87 (t, *J* = 6.5 Hz, 3H, H-15′); ^13^C-NMR (CDCl_3_, 75.47 MHz) δ: 146.3 (C-2), 143.4 (C-1), 128.8 (C-6), 122.3 (C-5), 119.1 (C-4), 108.1 (C-3), 55.9 (2-OCH_3_), 31.9 (C-2′), 30.9 (C-1′), 37.2 (C-2′), 29.8–22.7 (C-2′-14′), 14.1 (C-15′).

#### 3.1.3. Synthesis of 2-methoxy-6-pentadecyl-1,4-benzoquinone (**9**)

A solution of **8** (2.17 g, 6 mmol) in DMF was bubbled with O_2_ for 5 min and, after the O_2_ atmosphere, was kept over the reaction vessel. Salcomine was added (380 mg, 1.17 mmol), and the reaction was stirred at r.t. for 24 h. After, diethyl ether (20 mL) was added, and the organic layer was washed with 0.1 M HCl (2 × 10 mL), water (10 mL) and brine (10 mL). The organic layer was dried over anhydrous sodium sulfate, and the solvent evaporated. The obtained solid was purified by crystallization from petroleum ether 40–60 °C, affording the title compound (**9**) (1.85 g, 82%) as a yellow solid.

*2-methoxy-6-pentadecyl-1,4-benzoquinone* (**9**): ^1^H-NMR (CDCl_3_, 300.13 MHz): δ = 6.49–6.47 (m, 1H, H-5), 5.87 (d, *J* = 2.4 Hz, 1H, H-3), 3.82 (s, 3H, 2-OCH_3_), 2.44 (ddd, J = 7.5 Hz, 1.4, 2H, H-1′), 1.54–1.45 (m, 2H, H-2′), 1.30–1.25 (m, 24 H, H-3′-14′), 0.88 (t, *J*= 6.5, 3H, H-15′); ^13^C-NMR (CDCl_3_, 75.47 MHz) δ: 187.7 (4-CO), 182.2 (1-CO), 158.9 (C-2), 147.6 (C-6), 132.9 (C-5), 107.1 (C-3), 55.3 (2-OCH_3_), 28.7 (C-1′), 27.7 (C-2′), 29.8–22.7 (C-2′-14′), 14.1 (C-15′).

#### 3.1.4. Synthesis of 2-methoxy-6-pentadecylbenzene-1,4-diol (**10**).

Compound **9** (1.86 g, 0.005 mol) was dissolved in chloroform and added to a 10% solution of Na_2_S_2_O_4_ in hot water. The mixture was shaken for 10 min, and, after the aqueous layer was drawn off, the chloroform was shaken with brine, dried over anhydrous sodium sulfate, and the solvent evaporated under reduced pressure. The residue was purified by crystallization from *n*-hexane, affording the title compound (**10**) (1.31 g, 87%) as a white solid.

*2-methoxy-6-pentadecylbenzene-1,4-diol* (**10**): m.p. (*n*-hexane) = 92–94 °C; ^1^H-NMR (CDCl_3_, 300.13 MHz): ^1^H-NMR (CDCl_3_, 300.13 MHz): δ 6.31 (d, *J* = 2.8 Hz, 1H, H-3), 6.20 (d, *J* = 2.8 Hz, 1H, H-5), 3.84 (s, 3H, 2-OCH_3_), 2.56 (t, *J* = 7.5 Hz, 2H, H-1′), 1.60–1.52 (m, 2H, H-2′), 1.30–1.25 (m, 24H, H-3′-14′), 0.87 (t, *J* = 6.5 Hz, 3H, H-15′); ^13^C-NMR (CDCl_3_, 75.47 MHz): δ 148.3 (C-2), 147.6 (C-4), 137.3 (C-1), 107.9 (C-5), 97.0 (C-3), 56.3 (2-OCH_3_), 31.9 (C-2′), 30.9 (C-1′), 29.8–22.7 (C-2′-14′), 14.1 (C-15′); ESI-TOF-HRMS (+) *m*/*z*: Anal. Calc. for C_24_H_37_O_3_ (M+H^+^): 349.27320; found: 349.27372.

### 3.2. Biological Activity

#### 3.2.1. Human Cell Lines and Growth Conditions

Human breast adenocarcinoma MDA-MB-231, SK-BR-3, MDA-MB-468, melanoma A375, hepatocellular carcinoma Huh-7, colorectal carcinoma HCT116, and non-tumorigenic breast epithelial MCF10A cell lines were purchased from ATCC (Rockville, MD, USA). Cancer cells were cultured in RPMI-1640 medium with UltraGlutamine (Biowest, VWR, Carnaxide, Portugal) with 10% fetal bovine serum (FBS; Gibco, Alfagene, Lisboa, Portugal). MCF10a cells were cultured in DMEM:F12 with 10% FBS or MEGM SingleQuot Kit Suppl. & Growth Factors, respectively (Lonza, VWR, Carnaxide, Portugal). Cells were maintained at 37 °C in a humidified atmosphere of 5% CO2. Mycoplasma detection was routinely performed using the MycoAlert™ PLUS kit (Lonza).

#### 3.2.2. Sulforhodamine B (SRB) Assay

Human cell lines were incubated in 96-well plates at a final density of 5.0 × 10^3^ cells/well (for A375, Huh-7 and HCT116 cells), 1.0 × 10^4^ cells/well (for MDA-MB-231 and MCF10A) and 7.5 × 10^3^ cells/well (for SKBR3 and MDA-MB-468 cells) for 24 h. Cells were then treated with serial dilutions (0.313 µM to 50 µM) of compounds for 48 h. The effect of these compounds on in vitro growth was assessed using the SRB assay. Cells were fixated with 12.5% TCA for 1 h at 4 °C. Plates were then stained with 0.25% SRB (Sigma-Aldrich, Sintra, Portugal) and washed with 1% acetic acid. The bound dye was solubilized in 0.12% Tris Base and the absorbance was measured at 510 nm in a microplate reader (Biotek Instruments Inc., Synergy, MX, USA). The maximum concentration of solvent used in this assay (1% DMSO) was included as control. The GI_50_ was then determined.

#### 3.2.3. Colony Formation Assay

MDA-MB-231 cells were seeded in 6-well plates at a density of 5.0 × 10^2^ cells/well, followed by incubation with hydroquinone **10** for 9 days. Formed colonies were fixed with 10% MeOH and 10% AcOH for 10 min, and then they were stained with 0.5% crystal violet (Sigma-Aldrich, Sintra, Portugal) in 1:1 MeOH/H_2_O for 15 min. Colonies containing more than 20 cells were counted.

#### 3.2.4. Cell Viability Assay

The percentage of viable cells was determined using the Trypan Blue Assay. Briefly, MDA-MB-231 cells were seeded in 24-well plates at 6 × 10^4^ cells/well for 24 h, followed by treatment with hydroquinone **10** for 24 h. Cells were then harvested and resuspended in 0.4% Trypan Blue (Sigma-Aldrich, Sintra, Portugal), and the number of viable/dead cells were counted using a Leica light optical microscope (Wetzlar). Vehicle (0.25% DMSO) was included as a control.

#### 3.2.5. Cell Cycle Analysis

Cell cycle and apoptosis were analyzed as described [16]. Briefly, MDA-MB-231 cells were incubated in 6-well plates at a density of 2.25 × 10^5^ cells/well for 24 h. Cells were then treated with hydroquinone **10** or DMSO only for 48 h. For cell cycle analysis, cells were stained with propidium iodide (PI; Sigma-Aldrich, Sintra, Portugal); cell cycle scattering was analyzed by flow cytometry and cell cycle phases were quantified using FlowJoX v10.0.7 (Treestar, Woodburn, OR, USA).

#### 3.2.6. Western Blot Analysis

MDA-MB-231 cells were seeded in 6-well plates at a density of 2.25 × 10^5^ cells/well for 24 h, followed by treatment with 2 µM hydroquinone **10**. Protein extracts were quantified using the Bradford reagent (Sigma-Aldrich, Sintra, Portugal). Proteins were run in SDS-PAGE and transferred to a Whatman nitrocellulose membrane from Protan (VWR, Carnaxide, Portugal). After blocking, proteins were identified using a mouse monoclonal anti-anti-MDM2 (D-12), anti-Bax (2D2), anti-Bcl-2 (C-2), or a rabbit polyclonal anti-p21 (C-19), followed by an anti-mouse or anti-rabbit horseradish-peroxidase (HRP)-conjugated secondary antibody. For the loading control, a mouse monoclonal anti-GAPDH (6C5) was used. The signal was detected with the ECL Amersham kit from GE Healthcare (VWR, Carnaxide, Portugal). The ChemiDoc™ XRS Imaging System from Bio-Rad Laboratories (Amadora, Portugal) for Microsoft Windows 10 was used for detection.

#### 3.2.7. Statistical analysis

Data were statistically analyzed using GraphPad Prism version 7.0. Different statistical tests were used accordingly to the dataset; *p* values <0.05 or < 0.001 were considered statistically significant.

## 4. Conclusions

In this work, the demethylated derivative of hierridin B (**6**) and norhieridin B (**10**), as well as its structurally related quinone (**9**), were synthesized for the first time. The study of their effect on the in vitro cell growth inhibition of human tumor cell lines revealed that norhierridin B (**10**) was the most effective in all cancer cell lines, exhibiting much lower IC_50_ values than its structurally related quinone (**9**) and hierridin B (**6**). Particularly, hydroquinone **10** exhibited a noticeable growth inhibitory activity against the TNBC cells MDA-MB-231, SKBR3, and MDA-MB-468, with these being effects associated with cell cycle arrest; increase of p21 and Bax; and decrease of Bcl-2 protein expression levels. The interference of hydroquinone **10** in MDA-MB-231 cells with the protein expression levels of the p53 transcriptional targets p21, Bax, Bcl-2, and MDM2 suggests the activation of a p53 pathway, which should be further explored in the future. Altogether, these results indicate that hydroquinone **10** could be an effective cytotoxic agent against TNBC cells, which should be further explored in the future.

## Figures and Tables

**Table 1:** Effect of compounds **6**–**10** on the growth of human cancer cell lines.

	GI_50_ (μM)					
Cell line/Compound	6	7	8	9	10	Etoposide
MDA-MB-231	>50	9.65 ± 0.15	28.8 ± 3.3	5.83 ± 1.10	0.61 ± 0.07	5.06 ± 1.24
SKBR3	>50	14.0 ± 0.0	>50	4.35 ± 0.15	0.77 ± 0.06	4.77 ± 1.09
MDA-MB-468	>50	7.85 ± 2.15	31.00 ± 7.00	6.65 ± 0.90	0.68 ± 0.13	2.14 ± 0.36
A375	>50	16.0 ± 1.7	32.9 ± 4.2	20.6 ± 1.9	2.0 ± 0.4	0.9 ± 0.07
Huh-7	>50	27.5 ± 0.5	>50	2.95 ± 0.15	0.61 ± 0.03	3.43 ± 0.85
HCT116	>50	26.5 ± 0.5	26.0 ± 3.2	29.6 ± 0.5	3.2 ± 0.6	0.67 ± 0.05

GI_50_ was determined by sulforhodamine (SRB) assay after 48 h treatment (growth obtained with vehicle was set as 100%). Data are mean ± SEM of two to five independent experiments. Etoposide was used as positive control.

## Supplementary Materials

The following are available online, Figure S1: ^1^H and ^13^C-NMR of compound **7**, Figure S2: ^1^H and ^13^C-NMR of compound **8**, Figure S3: ^1^H and ^13^C-NMR of compound **9**, Figure S4: ^1^H and ^13^C-NMR of compound **10**, Figure S5: HRMS of compound **10**.

## References

[1] García P., Hernández Á., San Feliciano A., Castro M. (2018). Bioactive Prenyl-and Terpenyl-Quinones/Hydroquinones of Marine Origin. Mar. Drugs..

[2] Asche C. (2005). Antitumour quinones. Mini Rev. Med. Chem..

[3] Sunassee S.N., Davies-Coleman M.T. (2012). Cytotoxic and antioxidant marine prenylated quinones and hydroquinones. Nat. Prod. Rep..

[4] Chang H.-S., Lin Y.-J., Lee S.-J., Yang C.-W., Lin W.-Y., Tsai I.-L., Chen I.-S. (2009). Cytotoxic alkyl benzoquinones and alkyl phenols from Ardisia virens. Phytochemistry..

[5] Cheng-Sánchez I., Torres-Vargas J.A., Martínez-Poveda B., Guerrero-Vásquez G.A., Medina M.Á., Sarabia F., Quesada A.R. (2019). Synthesis and Antitumor Activity Evaluation of Compounds Based on Toluquinol. Mar. Drugs..

[6] Leao P.N., Costa M., Ramos V., Pereira A.R., Fernandes V.C., Domingues V.F., Gerwick W.H., Vasconcelos V.M., Martins R. (2013). Antitumor activity of hierridin B, a cyanobacterial secondary metabolite found in both filamentous and unicellular marine strains. PloS ONE..

[7] Gonzalez A.G., Barrera J.B., Perez E.M.R. (1992). Synthesis of hierridin, a phenol from the lichen, Ramalina hierrensis. Phytochemistry..

[8] Liu X., Lu G., Guo Y., Guo Y., Wang Y., Wang X. (2006). Catalytic transfer hydrogenolysis of 2-phenyl-2-propanol over palladium supported on activated carbon. J. Mol. Catal. A: Chem..

[9] Feng J., Yang C., Zhang D., Wang J., Fu H., Chen H., Li X. (2009). Catalytic transfer hydrogenolysis of α-methylbenzyl alcohol using palladium catalysts and formic acid. Appl. Catal. A: Gen..

[10] Sawadjoon S., Lundstedt A., Samec J.S. (2013). Pd-catalyzed transfer hydrogenolysis of primary, secondary, and tertiary benzylic alcohols by formic acid: A mechanistic study. Acs Catal..

[11] König W.A., Faasch H., Heitsch H., Colberg C., Hausen B.M. (1993). Synthese von seitenkettenmodifizierten Analogen des Allergens Primin/Synthesis of Side-Chain-Modified Analogues of the Allergen Primin. Z. Naturforsch. B..

[12] Davis C.J., Hurst T.E., Jacob A.M., Moody C.J. (2005). Microwave-mediated Claisen rearrangement followed by phenol oxidation: A simple route to naturally occurring 1, 4-benzoquinones. The first syntheses of verapliquinones A and B and panicein A. J. Org. Chem..

[13] Costa M., Sampaio-Dias I.E., Castelo-Branco R., Scharfenstein H., Rezende de Castro R., Silva A., Schneider M.P.C., Araújo M.J.O., Martins R.R., Domingues V.F. (2019). Structure of Hierridin C, Synthesis of Hierridins B and C, and Evidence for Prevalent Alkylresorcinol Biosynthesis in Picocyanobacteria. J. Nat. Prod..

[14] Mehanna J., Haddad F.G., Eid R., Lambertini M., Kourie H.R. (2019). Triple-negative breast cancer: Current perspective on the evolving therapeutic landscape. Int. J. Womens Health..

[15] Perrin D., Armarego W. (1988). Purification of Organic Chemicals.

[16] Soares J., Espadinha M., Raimundo L., Ramos H., Gomes A.S., Gomes S., Loureiro J.B., Inga A., Reis F., Gomes C. (2017). DIMP 53-1: A novel small-molecule dual inhibitor of p53–MDM 2/X interactions with multifunctional p53-dependent anticancer properties. Mol. Oncol..

