# Supplementary material for: Norhierridin B, a New Hierridin B-Based Hydroquinone with Improved Antiproliferative Activity
Source: Molecules. 2020 Mar 30;25(7):1578. doi: 10.3390/molecules25071578 (PMC7181126; doi:10.3390/molecules25071578)
Supplement: Supplementary file 1 [file molecules-25-01578-s001.pdf]

SUPPORTING INFORMATION

Norhierridin B, a New Hierridin B-Based Hydroquinone with Improved Antiproliferative Activity

Pedro Brandão [1,#], Joana Moreira [1,2,#], Joana Almeida [3], Nair Nazareth [3], Ivo E. Sampaio-Dias [4], Vitor Vasconcelos [2,5], Rosário Martins [2,6], Pedro Leão [2], Madalena Pinto [1,2], Lucília Saraiva [3,*], Honorina Cidade [1,2,*]

1 Laboratory of Organic and Pharmaceutical Chemistry, Department of Chemical Sciences, Faculty of Pharmacy, University of Porto, Rua de Jorge Viterbo Ferreira 228, 4050-313 Porto, Portugal; pedrocgbrandao@gmail.com (P.B.); joana.m26@hotmail.com (J.M.); madalena@ff.up.pt (M.P.)

2 CIIMAR/CIMAR, Interdisciplinary Centre of Marine and Environmental Research, University of Porto, Edifício do Terminal de Cruzeiros do Porto de Leixões, Avenida General Norton de Matos, S/N, 4450-208 Matosinhos, Portugal; pleao@ciimar.up.pt (P.L.)

3 LAQV/REQUIMTE, Laboratory of Microbiology, Department of Biological Sciences, Faculty of Pharmacy, University of Porto, Rua Jorge Viterbo Ferreira, 228, 4050-313 Porto, Portugal; naircampos@gmail.com (N.N.); JoanaAlmeida15@gmail.com (J.A.)

4 LAQV/REQUIMTE, Department of Chemistry and Biochemistry, Faculty of Sciences, University of Porto, Rua do Campo Alegre 687, 4169-007 Porto, Portugal; idias@fc.up.pt (I.E.S.-D.)

5 Department of Biology, Faculty of Sciences, University of Porto, Rua do Campo Alegre, Edifício FC4, 4169-007 Porto, Portugal; vmvascon@fc.up.pt (V.V.)

6 Health and Environment Research Centre, School of Health, Polytechnic Institute of Porto, Rua Dr. António Bernardino de Almeida, 400 ,4200-072 Porto, Portugal; mrfmarti@gmail.com (R.S.)

[#] Authors contributed equally to this work.

Correspondence: lucilia.saraiva@ff.up.pt (L.S.); hcidade@ff.up.pt (H.C.); Tel.: +351-220428584 (L.S.); +351-220428688 (H.C.)

NMR spectra

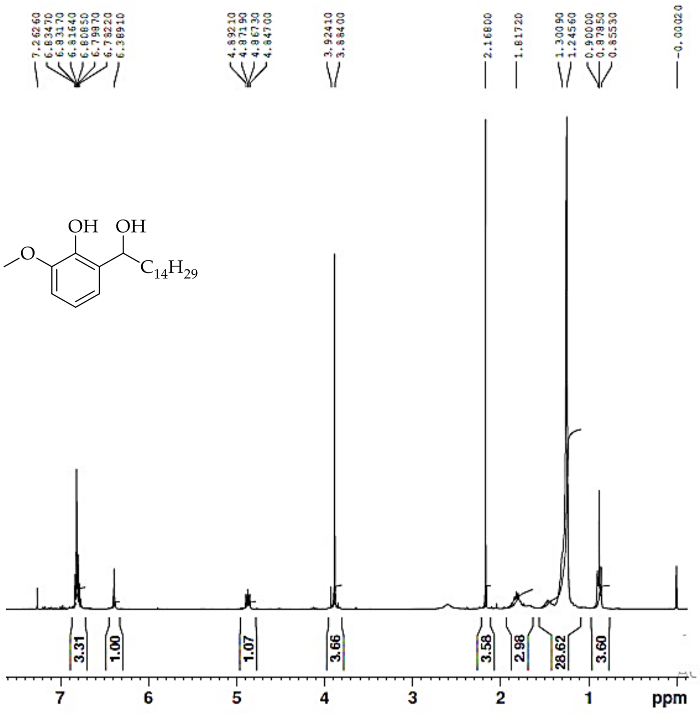

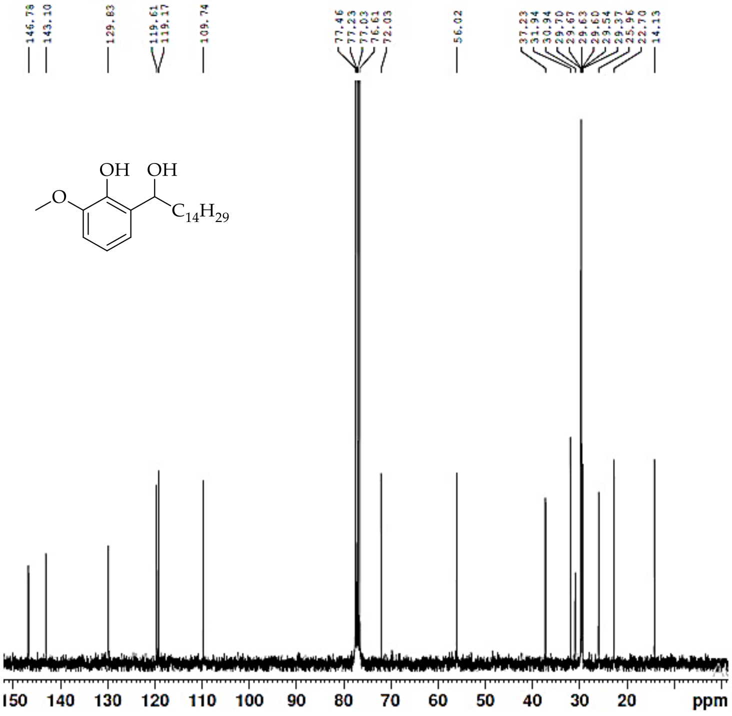

Figure S 1. ^{1}H and ^{13}C NMR of compound **7**.

NMR spectra

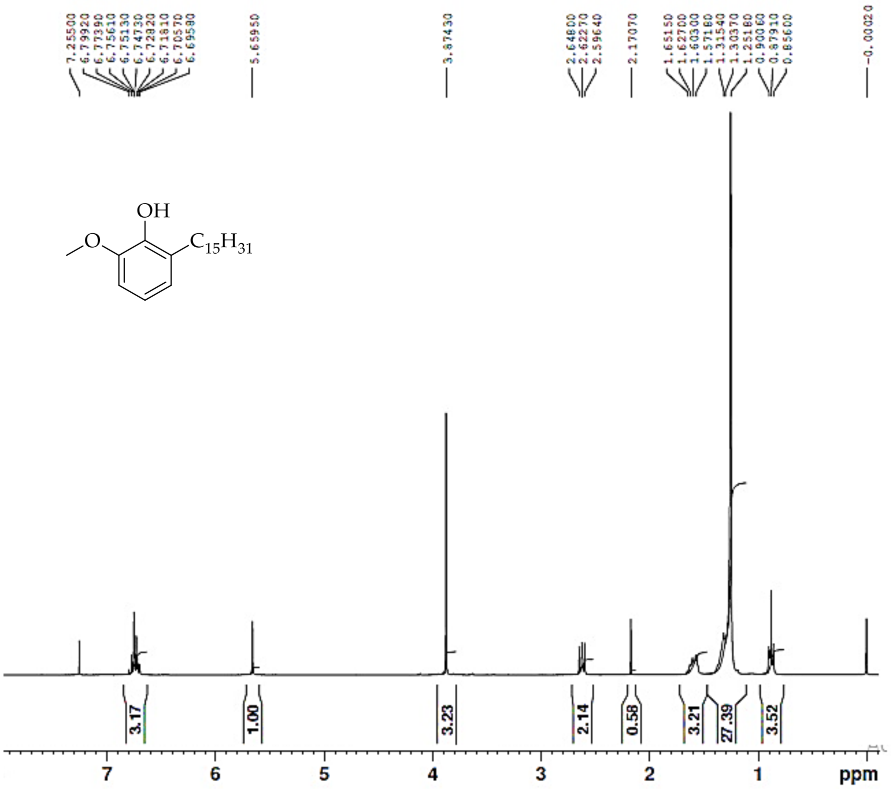

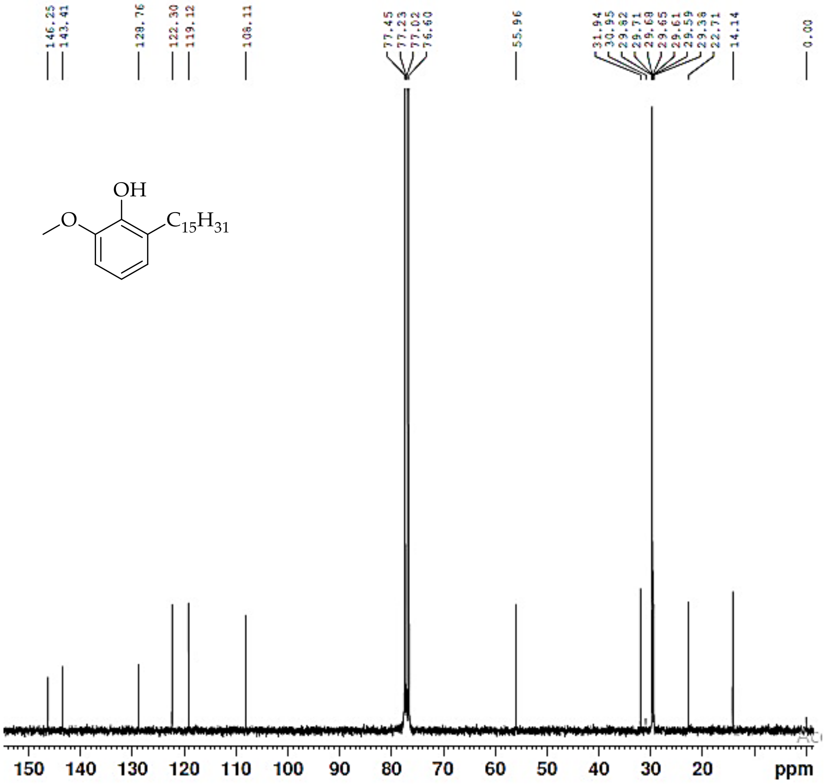

Figure S 2. ^{1}H and ^{13}C NMR of compound **8**.

NMR spectra

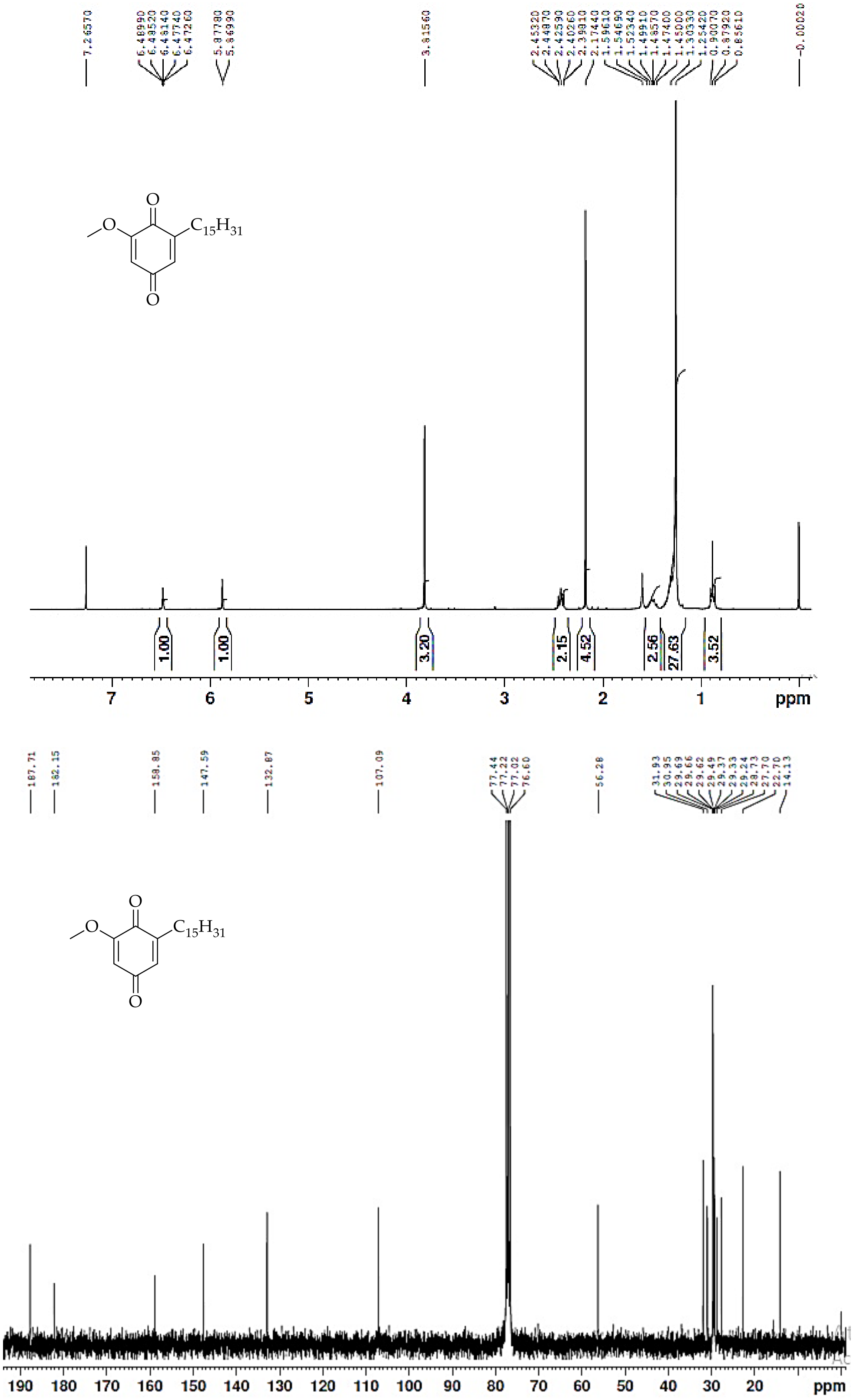

Figure S 3. ^{1}H and ^{13}C NMR of compound **9**.

NMR spectra

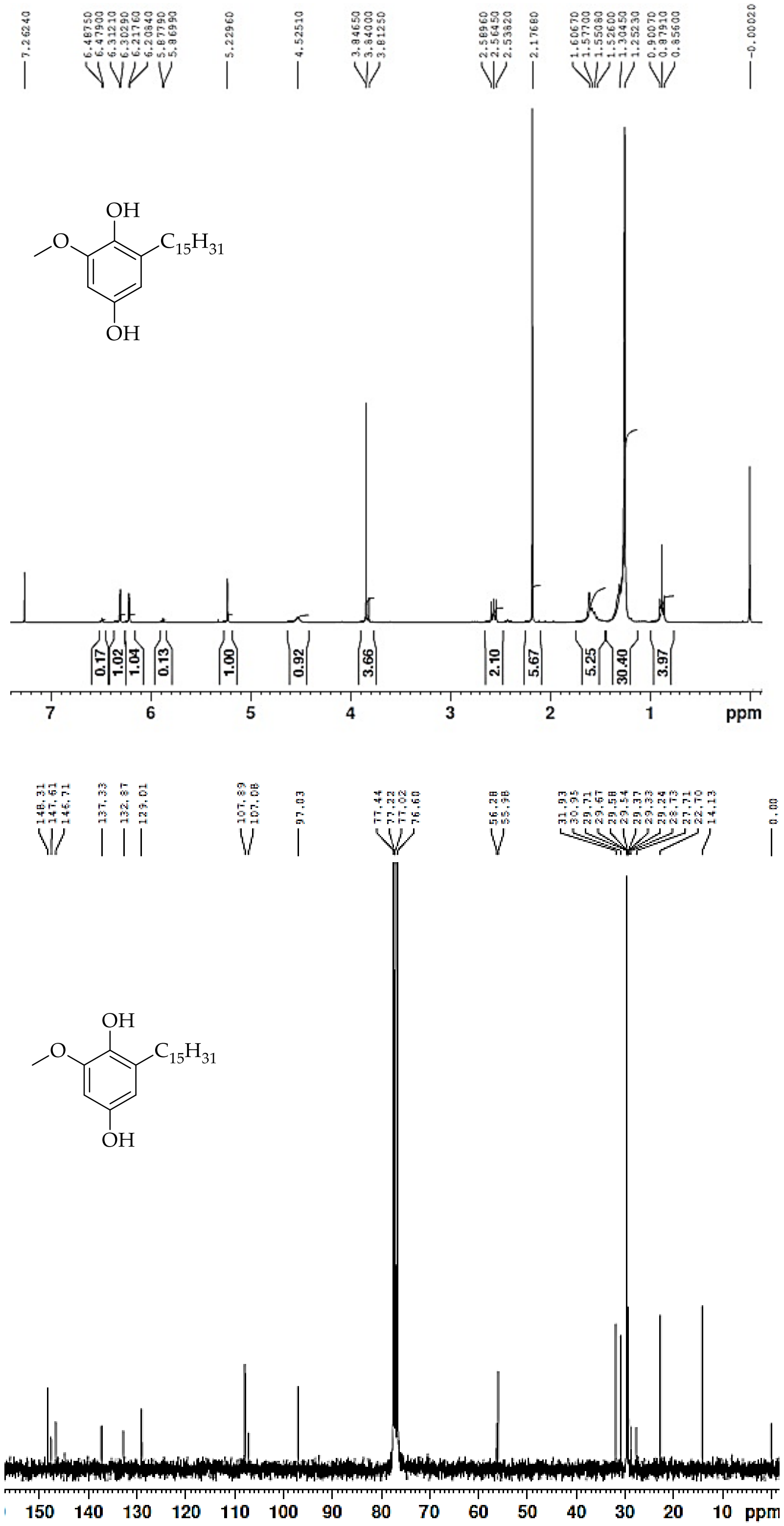

Figure S 4. ^{1}H and ^{13}C NMR of compound **10**.

Mass Spectrum List Report

Analysis Info **Electrospray (ESI)**
Analysis Name JRHC1600074_000001.d
Sample Name
Acquisition Date 1/14/2016 10:36:17 AM
Instrument apex-Qe

Acquisition Parameter
Capillary Exit 300.0 V
Skimmer 1 20.0 V

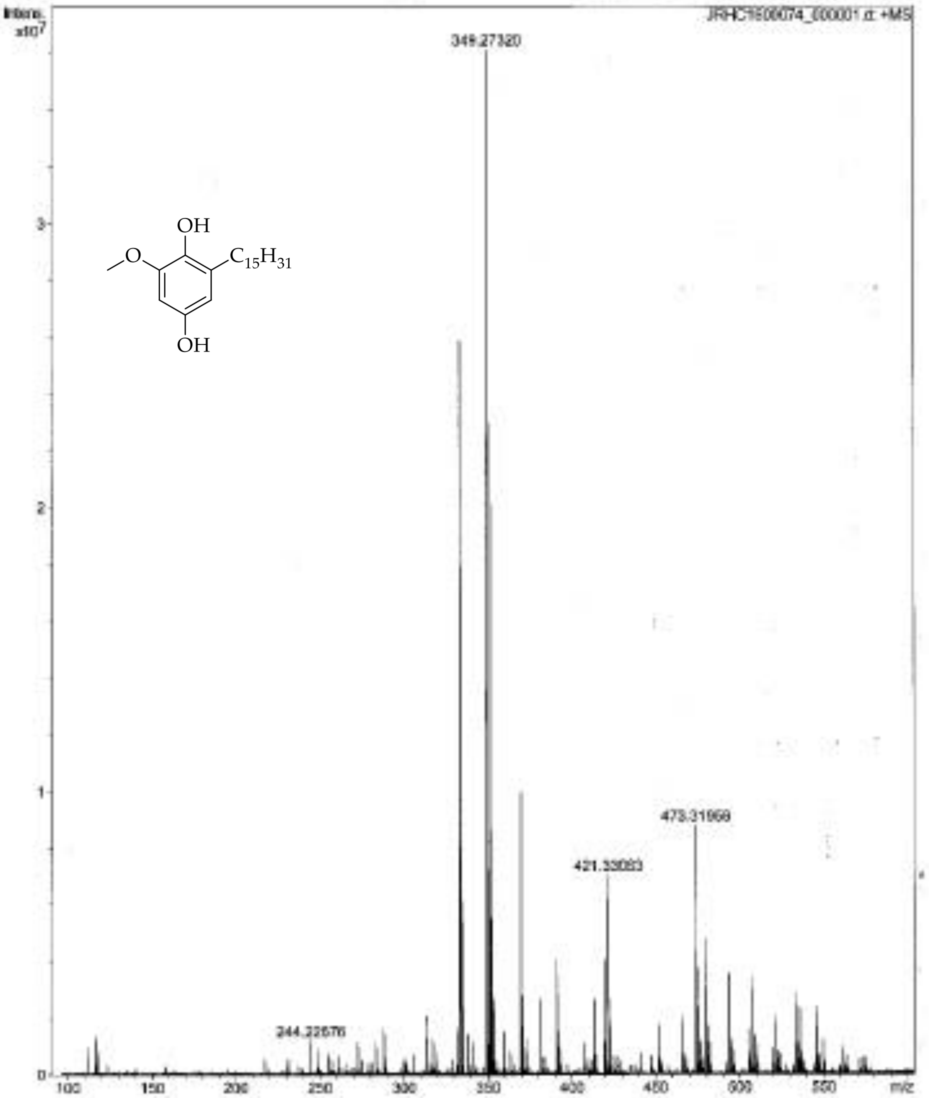

Mass Spectrum Molecular Formula Report

Meas. m/z	#	Formula	Score	m/z	err [mDa]	err [ppm]	mSigma	rdb	e⁻ Conf	N-Rule
349.27320	1	C 22 H 37 O 3	100.00	349.27372	0.52	1.49	20.6	4.5	even	ok
351.28888	1	C 22 H 39 O 3	100.00	351.28937	0.49	1.39	19.8	3.5	even	ok

Figure S 5. HRMS of compound **10**.
